# A Re-Evaluation of Olive Fruit Fly Organophosphate-Resistant *Ace* Alleles in Iberia, and Field-Testing Population Effects after in-Practice Dimethoate Use

**DOI:** 10.3390/insects10080232

**Published:** 2019-08-01

**Authors:** Tânia Nobre, Luis Gomes, Fernando Trindade Rei

**Affiliations:** Laboratory of Entomology, ICAAM—Instituto de Ciências Agrárias e Ambientais Mediterrânicas, Universidade de Évora, 7006-554 Évora, Portugal

**Keywords:** dimethoate, *Bactrocera oleae*, resistance, organophosphate, acetylcholinesterase, in-practice field test

## Abstract

The management of the olive fruit fly (*Bactrocera oleae*) is traditionally based upon the use of organophosphate insecticides, mainly dimethoate. In this evolutionary arms race between man and pest, the flies have adapted a pesticide resistance, implying two point-mutations of the *Ace* gene -I214V and G488S- and a 9bp deletion -Δ3Q. We revisited 11 Iberian locations to evaluate this adaptation of organophosphate (OP)-resistant alleles through amplicon sequencing. Screening for populations where the wild type is prevalent allows an identification of hotspots for targeted mitigation measures; we have hence refined the scale to the region with the lowest OP-resistant alleles frequency 71 locations were sampled and individuals checked using a fast and low-cost allele-specific-primer polymerase chain reaction (ASP-PCR) method]. An increase in *Ace* gene point-mutations was observed, and the Δ3Q mutation remains undetected. The lowest frequencies of the OP-resistant alleles remain in the west, underlining the hypothesis of an introduction of resistance from eastern Mediterranean areas. A field test was performed by sampling the fly population before and after in-practice dimethoate application. A clear reduction in olive fruit fly numbers was observed, with no relevant changes in the genotypic frequencies of the resistance alleles. The findings are discussed in frame of the type and intensity of the selection pressure that has led to the adaptation to resistance and its consequences from the producer perspective.

## 1. Introduction

In the evolutionary arms race [1] against agricultural pests, the intensive use of organophosphates (OPs) in the last 50 years has led to an evolution (or adaptation) of resistance. Insensitivity to these insecticides by mutations of the *Ace* gene, which codes for acetylcholinesterase, the target enzyme of OPs, has been demonstrated in several insects, including mosquito species, in *Drosophila melanogaster* (common fruit fly), *Musca domestica* (common housefly), *Ceratitis capitata* (Mediterranean fruit fly), *Aphis gossypii* (the aphid) and *Bactrocera oleae* [2,3,4,5,6]. In most insects, two types of *Ace* genes (*ace1* and *ace2*) have been identified [7,8]. However, in some Diptera including tephritids (such as the *Bactrocera* species), the *ace1* gene is absent, likely due to gene loss, and only a single *ace2* gene has been identified [9].

Pesticide use applies an exceptionally specific selective pressure, and this strong selection pressure may lead to the evolution of resistance. Resistance can evolve through selection from standing variation or through ‘de novo’ mutations. 

Particularly in the cases where a specific pesticide-binding site is involved, selection can be target-specific, acting upon a single molecular target, and sometimes even in a specific binding residue. In this situation, ‘de novo’ mutations might be more prone to occur, even though limited by the constraints imposed by target protein functionality. Evidence supporting these initial assumptions comes, for example, from studies on mosquitoes on target-site resistance to pyrethroids, and to organophosphates (OPs) and carbamates (CMs) [10,11]. Such a targeted selection pressure can result in parallel ‘de novo’ mutations, which repeatability depends upon the functional constraints affecting each target site and other possible resistance mechanisms [12].

In the particular case of *Bactrocera oleae* (Rossi, 1790), resistance to OPs likely evolved by ‘de novo’ mutations at the *Ace* gene. Over the last decades, the management of the olive fruit fly has been based on the use of OPs, mainly dimethoate. The first record of *B. oleae* resistance to dimethoate dates back to the 1970s [13,14], but it was only 30 years later that the mechanism started to be studied and understood [6,15]. Two point-mutations were first identified in the *Ace* gene as the underlying cause of OP resistance in the olive fruit fly: I214V (nucleotide mutation at the position 214 from the reference AF452052 replaces an isoleucine by a valine) and G488S (nucleotide mutation at the position 488 from the reference AF452052 replaces a glycine by a serine). These changes affect amino-acids close to the active site of the enzyme, possibly causing topological changes that decrease the effectiveness of the action of the pesticide [6].

The presence of OP-resistant olive fruit flies in Europe has increased and has become widespread [16,17,18,19]. Supporting the hypothesis of parallel ‘de novo’ resistance-associated mutations, identical mutations were found in the oriental fruit fly, *Bactrocera dorsalis* [20] and I214V is the homolog to the I199V mutation found earlier in *Drosophila melanogaster* [21]. The general assumption for ‘de novo’ resistance evolution is that mutation continues at the background rate, and it is the highly specific selective pressure imposed by the pesticide that leads to an increase in the frequency of the mutations conferring resistance, balanced by any associated fitness costs [12]. Skouras and co-workers [22] accessed the resistance of olive fruit fly populations in Greece and Cyprus through the topical application of dimethoate, and showed the presence of both point mutations in homozygocity in the least resistant individuals; this supports the importance of these mutations at a minimum level of resistance, but also suggests a low fitness cost.

A third mutation was detected, lying outside the active site gorge of AChE: A 9bp (GCAACAACA) deletion corresponding to the nucleotides 1926–1934 of the reference AF452052, and that results in a deletion of three glutamine residues at positions 642–644 inclusive (referred as a Δ3Q mutation) [23]. This mutation appears associated with higher OP doses resistance, and always in combination with the two point mutations, suggesting an auxiliary and/or multiplicative role of this mutation, in addition to its inability to alone provide a basal level of resistance [17]. Found originally in Greek populations, this mutation shows currently the highest frequencies in Greece and Italy and a gradual decrease of Δ3Q frequency is observed towards the western Mediterranean [17]. Whereas I214V and G488S mutations were detected in Portugal, at the Western end of the Mediterranean basin [18], Δ3Q was never found in this region [17,18]. The data thus suggest a spreading of the variants of the resistance gene from East within the Iberian Peninsula, where the frequency of the *Ace* OP-resistant alleles was reported to vary between 0.20 and 0.90 [18]. We revisited those locations to assess the evolution of *Ace* OP-resistant alleles. The region with the lowest frequency of resistance alleles reported corresponded to the Alentejo region in Portugal. Therefore, we have refined the sampling scale in this region, and screened the populations for *Ace* OP-resistant alleles, following the hypothesis that there has been an increase of the mutated alleles in the population, as the establishment of olive orchards under intensive and super intensive regimes has been observed in the region. To understand the practical consequences of the resistance, from the producer perspective, we have put together a test where the *B. oleae* population was screened before and after a dimethoate application done in the field, in a classic production system, and according to the common field practices. 

## 2. Materials and Methods 

### 2.1. Olive Fly Samples

**Figure 1:**
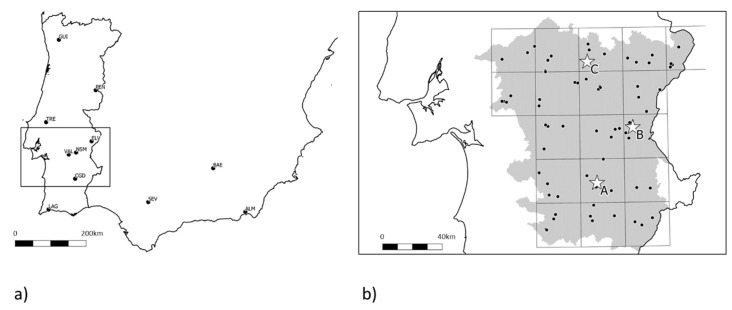
Sample collection points. (**a**) Sampling set I (October/November 2016), revisiting the localities sampled in [18]; (**b**) Sampling set II (October/November 2017), comprehensive sampling in the region Alentejo (dots), where the lowest frequency of the resistance allele was found; in-practice field test locations (stars) (October/November 2017).

The sample collection for monitoring insect resistance was structured in a two-step process. First, olive fruit flies were collected revisiting the localities sampled in [18], of which nine were in Portugal and four in Andalusia, Spain (Figure 1a—Sampling set I). Secondly, a comprehensive sampling effort took place in the region of Alentejo, Portugal, as it corresponded to the sampling location with lower frequency of the resistance allele according to the same study. In this case, a stratified random sampling was designed to cover the region: A grid of 30 × 30 km in a total of 18 squares comprised the stratification of the sampling, and inside each square up to four olive areas were randomly selected (depending upon availability), where fruits with signs of oviposition by *B. oleae* were collected whenever available. It was possible to obtain biological material from 71 locations (Figure 1b—Sampling set II), being a square grid represented on average by 20 genotypes. In all cases, these sampled trees were not in olive groves of intense production, and were taken in abandoned olive groves, confirmed organic olive groves, or in decorative specimens, as a way to guarantee that no dimethoate had been applied directly in recent years. Olives were collected and stored in plastic boxes, with emerging larvae, pupae and adults being gathered periodically. From the olives collected in Malaga (Spain) and Paradela (Portugal), no flies emerged. 

Individuals were stored at −20 °C in 70% ethanol until DNA extraction. From sampling set I, we target to analyze 10 individuals per sampling location (from Baeza, only five flies emerged) as done in [18], and for comparison purposes. From sampling set II, aimed to evaluate the olive fruit fly resistance region, we targeted for 20 individuals per grid square (five individuals per location; but 6 points had four, 4 with three, and 3 with one individuals). Individual flies were allowed to dry on filter paper prior to DNA extraction. DNA from the whole body tissue was extracted following extraction protocols using CTAB extraction buffer [24] after being grinded with a plastic pestle. Proteins were removed with 24:1 isoamyl alcohol:chloroform, and DNA precipitated with isopropanol. DNA extracts were eluted in 50 μL of sterile water. All extraction products were stored at −20 °C, and were later used directly in the PCR. 

### 2.2. Resistance Alleles Identification

Targeting the two resistance-associated *BoAce* mutations previously identified in the region- I214V and G488S-, we followed the procedure described in [16] to identify the resistance allele in the first sample set (Portugal and Andalusia, sampling set I). The primer pair BoAce_518F: TACTCAATTTCACTTTCAGCACTC and BoAce_1040R: CAACTCACCGACAATAGCG was used to amplify the 521 bp fragment in exon III, where the SNP known to be responsible for the I214V mutation is found; and the primer pair BoAce_1424F: CAGCTGGGTTGGTAATCC and BoAce_1519R: TAGTGCACGGAAGCTCC was used to amplify the 94 bp fragment in exon VI, which includes the SNP leading to G488S mutation. PCR reactions were conducted using 1 μL of the extracted DNA in a standard 25 μL reaction, with 0.5 pmol/μL of each primer, 1.5 mM MgCl_2_, 0.5 mM dNTPs and 0.05 U/mL Taq DNA polymerase. The cycle protocol involved initial denaturation at 94 °C for 2 min, followed by 35 cycles of 94 °C for 30 s, 55 °C for 30 s and 72 °C for 1 min, and an extension cycle of 72 °C for 7 min; the PCR product was purified using the NZYGelpure kit (from NZYTech, Lda, Lisboa, Portugal), and sequencing was done commercially (Macrogen Inc. Seoul, South Korea) on both strands. Analogous to the procedure in other works checking for codominant single nucleotide polymorphisms (SNPs), we looked for double peaks with a similar height in the sequence chromatograms [25]. Haplotype determinations in PCR products with heterozygosity at the targeted positions were made by an amplification with allele-specific primers designed as follows:

(a) For I214V, the allele-specific forward primer was designed so that its 3’ terminal nucleotide corresponds to the target SNP, and a single nucleotide artificial mismatch was introduced at the third nucleotide from the 3’ end of the primer to improve the specificity of allele-specific amplification (IV214_A 5′-CCGGCACTGCCACACTGGCTA-3′ and IV214_G 5′-CCGGCACTGCCACACTGGCTG-3′); the reversed primer used was non-allele specific (IV214_CP 5′-CCTCCTCGTAGCCCGGCA-3′).

(b) For G488S, and due to close by SNPs (within ten nucleotides 5’ to the target point mutation), the approach was having an allele-specific reverse primer, designed using the same principles described above (G488S_RG 5′-GGCAGGTGAAGAARTGATCTCC-3′ and G488S_RA 5′-GGCAGGTGAAGAARTGATCTCT-3′); this primer was paired with the non-allele specific BoAce_1424F.

The allele-specific primer PCR (ASP-PCR) method was initially developed for an allele analysis of clinically significant mutations [26] based on the principle that ASPs preferentially trigger an amplification of the specific allele and the presence of the SNP can thus be detected as positive PCR amplification after electrophoresis. Amplification followed a touch-down cycling protocol (TD-PCR): 95 °C for 3 min; a phase one of 10 cycles of 95 °C for 30 s, T_a_ for 30 s and 72 °C for 30 s; and 72 °C for 1 min, in which T_a_ was set to 65 °C, decreasing 1 °C per successive cycle until reaching the T_a_ of 55 °C; followed a phase two of 25 cycles using the final annealing temperature reached in the touchdown phase (T_a_ of 55 °C). After the amplification, electrophoresis was performed at 70 V for 45 min in 1×tris-acetate-EDTA buffer on 1.5% agarose gel stained with GreenSafe Premium (NzyTech) according to the protocol provided by the supplier. The amplified PCR products were visualized under a UV light, being a positive amplification indicative of the presence of the specific nucleotide tested at a given position (Figure S1). 

To test the capacity of the above-described allele-specific approach (ASP-PCR) in identifying the nucleotide(s) present at the targeted SNPs, we selected a sub-sample of the sampling set II comprising 5 individuals per grid (total of 85 specimens). This sub-sample was analyzed simultaneously by fragment sequencing and by ASP-PCR. Because the results that were obtained from the developed AS-PCR method were all consistent with the genotype data obtained using a direct DNA sequencing technique in 100% of the cases for the I214V and 98% for the G488S (being the failures due to failure of amplification, and hence no data result), we proceed to analyze the full sampling set II in this way. 

Searching for the Δ3Q variant was restricted to a sub-set of specimens in the revisited locations (sampling set I) using the allele-specific PCR protocol described in [17]. 

The frequency of the presence of resistance alleles from Pereira-Castro and co-workers [18] and the present study was compared by means of a Wilcoxon Signed-Ranks Test (α = 0.05). Also for data set II, the frequencies of the resistance alleles were calculated (20 genotypes per grid square) and an Empirical Bayesian Kriging (EKB) interpolation method, implemented in ArcGIS 10.1 Geostatistical Analyst, was used for data visualization [27].

### 2.3. In-Practice Field Test

To address, from the producer perspective, the impact of the *Ace* organophosphate (OP)-resistant alleles in the olive fly population, we designed a field test at three different traditional production orchards in the Alentejo region. We have sampled olives with signs of oviposition holes before and after the standard pesticide application procedure. The locations were chosen based on the frequency of the resistance allele found in the non-managed olive groves areas as described above (sampling set II). Per location, three boxes were assembled, each with 250 randomly collected, affected olives. The boxes were closed at all sides but two, where an insect net was glued to the sides; the mesh was small enough not to allow insects to get in or out, but big enough to allow ample air circulation. The boxes, left at room condition, were checked daily for larvae eclosion until no further insects emerged. The exact same procedure was repeated a week after the application of dimethoate at the standard allowed concentrations and procedures (1.2 L/ha of a product with 400 g/L of active substance). Because of no-independence of data sets, and the limitation of the trial to three locations, non-parametrical data dispersion measures of the number of larvae ecloding before and after the commercial application of dimethoate were plotted, highlighting the differences.

From each field, 10 randomly selected larvae were sequenced at the two fragments that include the resistance-associated *BoAce* mutations (procedure as above). The sequences were assembled, edited and the fragments were aligned using the software Unipro UGENE 1.32 [28] to look for variants and their frequencies, as well as the presence of other non-synonym mutations. 

## 3. Results

The presence of *Ace* OP-resistant alleles in the Western and Southern Iberian Peninsula had in general increased or stabilized, particularly in the cases where the frequency was already high (Table 1) and significant positive differences were found when comparing the previous sampling [18] and the one here reported (the Wilcoxon Signed-Ranks Test indicated that the median ranks in the present study -Mdn = 0.60- were significantly higher than the median ranks from the previous study -Mdn = 0.58; Z = 2.00, *p* < 0.05). The Alentejo region (where before the frequencies of *Ace* OP-resistant alleles were still relatively low), now faces frequencies equivalent to what was found in the rest of the Iberian Peninsula (Table 1), even when our sampling was done in abandoned and/or organic olive groves (and thus not subjected to the direct application of the pesticide). In none of the 105 Iberian flies screened did we detect the presence of the Δ3Q mutation.

**Figure 2:**
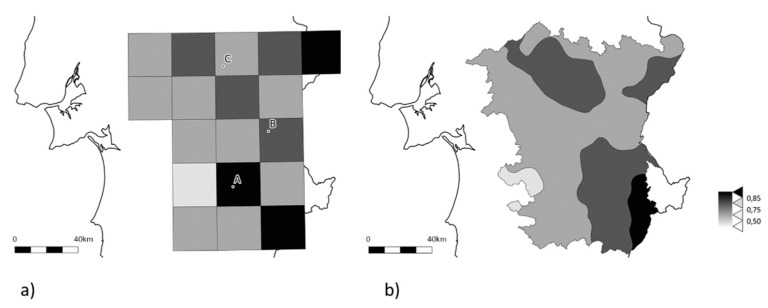
Schematic representation of OP resistance-associated alleles in the olive fruit fly based on 333 samples spread through 71 locations (see Figure 1b for details). (**a**) Observed frequencies by grid square; (**b**) Kriging estimated Alentejo region surface. The darker the gray, the greater is the proportion of OP resistance-associated alleles. White corresponds to the area with no data.

The developed AS-PCR method provides a fast screening of great number of samples, with an acceptable margin of error (the genotyping failures were due to an absence of amplification, and hence no data result), but it provides no further information on other regions of the gene of potential interest. However, for the aim of the work, this low-cost method allowed the screening of 333 samples, giving an overview of the frequency of the resistance allele (Figure 2a). The minimum frequency of the resistance alleles found was of 0.30, and the median value observed was of 0.70 (interquartile range (IQR) = 0.60–0.80); only 14 locations (corresponding to 20% of the sampled locations) had values below or equal to 0.50 (Table S1). The prediction of the frequencies of *Ace* OP-resistant alleles in the Alentejo region, done via EKB, suggests no clear hotspots where the wild type allele might still be prevalent in the population (Figure 2b), even though OP-resistance alleles still seem to be less widespread in the West of the Iberian Peninsula. 

**Figure 3:**
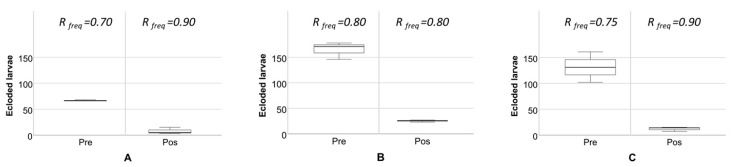
Boxplot of *Bactrocera oleae* larvae ecloding before and after the application of the dimethoate, highlighting the differences (site locations (**A**–**C**) on Figure 1b). R allele frequency before and after dimethoate application (details in Table S2).

The frequency of *B. oleae* eclosion from olive fruits with signs of oviposition (Figure 3) shows a clear decrease on eclosion rates after dimethoate application. In terms of the resistance genotypes detected from our in-practice field tests, both I214V and G488S were found at high levels in the population, before and after the application of dimethoate (Figure 3; Table S2). Indeed, the selected groves for the field tests showed generalized high frequencies of the resistance-alleles prior to application and not relating to the gradient found when analyzing the populations from the abandoned olive groves (Figure 2). 

## 4. Discussion

Our results support the previous observation of complete concordance between the zygosities of I214V and G488S in Iberian samples [18], strengthening the suggestion that, in this region, the resistance alleles result of a migration event of flies with a chromosome already carrying both substitutions, and are not a result of a local and independent ‘de novo’ origin. Insecticide resistance can spread through insect migration, and the speed of the process depends on migration rates. Crops provide a temporal and spatial concentration of resources, and olive groves cover wide areas of territory in the Iberian Peninsula, leading to elevated effective sizes of populations. 

We show an increase in the *Ace* OP-resistant allele’s frequencies in the studied region, likely following the conversion and establishment of olive groves under intensive and super intensive management regimes. The inclusion of dimethoate in the Priority List of Hazardous Substances (http://www.atsdr.cdc.gov/cercla), and the intention of discontinuing its use from the EU due to health risks, seem not yet to have practical results. Most of the olive groves areas are managed traditionally; dimethoate is authorized in most of the Mediterranean countries, including Spain, Italy, Greece and Portugal (http://ec.europa.eu/food/plant/pesticides/), and its use remains a common practice, although at least in Portugal, recently, only one application per year is permitted. In line with dimethoate use reduction, this active ingredient was already banned in French olive groves (in 2016). However, in such a general scenario, it seems that keeping a high population frequency of the OP-resistance alleles is still selected for. 

No data at the European level was found on the use of OPs specifically in olive groves, and as a surrogate we have looked for changes in olive crops fully converted or under conversion to organic farming. Eurostat data (https://ec.europa.eu/eurostat) is available for the time frame of 2012 to 2017, showing an increase of olive production under the organic farming regime (with the exception of Greece), with areas increase of 30% on average (albeit still low, in the total crop area). This tendency, to be kept and increased, should convert into a lower use of dimethoate, releasing the selection pressure on the olive flies’ populations. In such a scenario, it is hypothesized that the wild type alleles will increase in the population, being the rate depending on the fitness costs of these two point-mutations (and time for measurable shifts in the allele frequencies to be detected). 

As to the Δ3Q mutation, it remains undetected in the Iberian Peninsula. Whether there are indications that short insertions and deletions are frequent events, occurring in some genomes at a frequency that is comparable to the one of point mutations [29,30], in others the frequency seems to be lower [31,32]. This mutation was first found in Greek populations [23], and later its presence was confirmed widespread following the use of OP insecticides across the Mediterranean basin [17]. The most likely scenario is of a single origin of this mutation, and its spread results of the strong selective pressure imposed by the intensity of organophosphate use. However, at regular dose rates of OP, the progression of the Δ3Q mutation might be hindered by a higher fitness penalty. Because the level of resistance conferred by each variant, relative to the selecting dose of the pesticide, is also responsible for the variant selection [12], under a low OP pressure the mutations I214V and G488S are preferentially selected, increase in frequency and rapidly spread. Likely, the Δ3Q mutation will be selected for only whether the dose rate is high; in that situation the point-mutations alone (smaller-effect alleles) offer little selective advantage, as the pesticide will still be lethal to individuals carrying them. 

In the quest to find populations where the wild type is still prevalent, we made a fine screening around the region with the lowest frequency of OP-resistance alleles [18]. However, at those previously reported locations in the west of the Iberian Peninsula, if a change is to be recorded, it is the increase of OP-resistance associated *Ace* alleles. This underlines the hypothesis of an introduction of the resistance alleles from the more eastern Mediterranean areas into the Iberian Peninsula and its spreading to the west, where we could still find locations with frequencies below 0.4 (Table S1, Figure 2). These hotspots of “wild-type” *Ace* alleles are certainly of relevance once the selective pressure is released, and the non-mutated alleles might be able to recover their frequencies on the populations depending on the fitness penalty of the mutations that confer resistance. This has practical implications if effective measures for managing or containing resistance are to be put into place. 

As dimethoate keeps on being used to control populations of *B. oleae*, the resistance alleles will be fixated. In such a scenario the assumption was that no pest population suppression would be achieved at admissible doses of the active ingredient. However, the results of our field trial unexpectedly plea against the claimed dimethoate inefficiency on resistant populations. We have, at the moment, no explanation for the results observed, and only more questions were added. Is the dimethoate concentration allowed in Portugal higher than the level of resistance conferred by the two mutations observed in the active site gorge of *B. oleae*? Kakani and co-workers [33] suggested that these mutations may be the best combination possible—lowest fitness cost and highest resistance—to the organophosphates commonly used against olive fruit fly natural populations. But if it so, why is Δ3Q not yet detected? Is it just a question of time, or are the fitness costs in this context too high? 

The nature of our field test needs further consideration. Whereas in laboratory trials, tightly controlled research manipulating the particular factor under study is performed to determine the consequences of such manipulation, in field trials there is a complexity of uncontrolled variables. Particularly, the in-practice field test here performed was fully in a real-life context, as no variables were controlled: The dimethoate was applied in a production context, by three different operators (company workers) at the concentrations allowed. Other variables, not even reported, might be aiding the observed decrease in population numbers (e.g., high temperatures [34] or copper application [35]). The impact of operational control procedures, failures and their rates cannot be determined. In practice, the agronomists and respective production companies observe a marked decrease in population levels of olive fly as aimed, which will impair the needed decrease in dimethoate applications.

## 5. Conclusions

Olive fruit fly is arguably the most important olive tree pest with reported production losses up to 80% of oil value and 100% of some table cultivars [36]. Since the first record of olive fruit fly’s resistance to dimethoate in the Iberian Peninsula, we observed an increase in *Ace* gene point-mutations but the Δ3Q mutation remains undetected. Likely, the resistance alleles result of a migration event of flies with a chromosome already carrying both substitutions, and are not a result of a local and independent ‘de novo’ origin. The absence of Δ3Q mutation, in face of what is known on the dimethoate usage in traditional olive groves in the region, raises the question if the mutation fitness costs are too high in the present context or if it just a question of time. Hotspots where the wild type allele is still prevalent in the population are scarce but relevant once the selective pressure is released. Given the generalization of resistance, the side-effects on auxiliary-fauna, and the considerable economic impact of this insect, development of non-insecticidal pest management methods are highly desired, as the combination of non-chemical methods that may be individually less efficient than pesticides can generate valuable synergies.

## Figures and Tables

**Table 1:** Distributions of the I214V and G488S *Bactrocera oleae* Ace gene substitutions at sampling set I locations. S: Organophosphate (OP)-sensitive alleles (coding for 214I and 488G); R: OP-resistant alleles (coding for 214V and 488S). All samples were screened for Δ3Q.

Collection Site (Nearest Locality)	Code	Geographic Coordinates	Genotype			R Allele Frequency		
			SS/SS	SR/SR	RR/RR	Present Study	Pereira-Castro et al. [18]	Variation
Guimarães	GUI	41.46N; 8.31W	2	6	2	0.50	0.45	0.05
Penamacor	PEN	40.16N; 7.10W	1	2	7	0.80	0.75	0.05
Tremês	TRE	39.36N; 8.75W	0	3	7	0.85	0.55	0.30
Elvas	ELV	38.86N; 7.27W	1	6	3	0.60	0.55	0.05
N.S. de Machede	NSM	38.58N; 7.78W	3	4	3	0.50	0.40	0.10
Valverde	VAL	38.53N; 8.02W	2	7	1	0.45	0.20	0.25
Cabeça Gorda	CGD	37.91N; 7.82W	2	5	3	0.55	0.40	0.15
Lagos	LAG	37.13N; 8.68W	1	6	3	0.60	0.65	−0.05
Seville	SEV	37.27N; 5.50W	0	3	7	0.85	0.60	0.25
Baeza	BAE	38.05N; 3.37W	0	2	8	0.90	0.90	0.00
Almeria	ALM	36.89N; 2.44W	0	2	3	0.80	0.90	−0.10

## Supplementary Materials

The following are available online at https://www.mdpi.com/2075-4450/10/8/232/s1, Figure S1: Positive amplification is indicative of the presence of the specific nucleotide tested at a given position. See section ‘Methodology’ in the main text for details., Table S1: Distributions of the I214V and G488S *Bactrocera oleae* Ace gene substitutions at sampling set II. S: OP-sensitive alleles (coding for 214I and 488G); R: OP-resistant alleles (coding for 214V and 488S). Geographic coordinates in the system ETRS89/Portugal TM06, Table S2: Ecloded *Bactrocera oleae* larvae from 250 olive fruits with oviposition signs per olive grove replicate, and the presence of I214V and G488S *Bactrocera oleae* Ace gene substitutions before and after dimethoate application. S: OP-sensitive alleles (coding for 214I and 488G); R: OP-resistant alleles (coding for 214V and 488S). All samples were screened negative for Δ3Q.
