# Supplementary material for: A Re-Evaluation of Olive Fruit Fly Organophosphate-Resistant Ace Alleles in Iberia, and Field-Testing Population Effects after in-Practice Dimethoate Use
Source: Insects. 2019 Aug 1;10(8):232. doi: 10.3390/insects10080232 (PMC6723829; doi:10.3390/insects10080232)
Supplement: Supplementary file 1 [file insects-10-00232-s001.zip › Table S2.docx]

Table S2. Ecloded *Bactrocera oleae* larvae from 250 olive fruits with oviposition signs per olive grove replicate, and the presence of I214V and G488S Bactrocera oleae Ace gene substitutions before and after dimethoate application. S: OP-sensitive alleles (coding for 214I and 488G); R: OP-resistant alleles (coding for 214V and 488S). All samples were screened negative for Δ3Q.

| Olive grove | Geographic coordinates | **pre-dimethoate** | | | | | | **pos-dimethoate** | | | | | |
| --- | --- | --- | --- | --- | --- | --- | --- | --- | --- | --- | --- | --- | --- |
|  |  |  |  | Genotypes | | | R allele frequency |  |  | Genotypes | | | R allele frequency |
|  |  | larvae | rate | SS/SS | SR/SR | RR/RR |  | larvae | freq. | SS/SS | SR/SR | RR/RR |  |
| A | 38.18N; -7.75W | 66 | 0.26 |  |  |  |  | 3 | 0.01 |  |  |  |  |
|  |  | 66 | 0.26 | 1 | 4 | 5 | 0.70 | 5 | 0.02 | 0 | 2 | 8 | 0.90 |
|  |  | 68 | 0.27 |  |  |  |  | 15 | 0.06 |  |  |  |  |
| B | 38.52N; -7.53W | 171 | 0.68 |  |  |  |  | 23 | 0.09 |  |  |  |  |
|  |  | 178 | 0.71 | 0 | 4 | 6 | 0.80 | 27 | 0.11 | 0 | 4 | 6 | 0.80 |
|  |  | 146 | 0.58 |  |  |  |  | 26 | 0.10 |  |  |  |  |
| C | 38.93N; -7.82W | 102 | 0.41 |  |  |  |  | 7 | 0.03 |  |  |  |  |
|  |  | 131 | 0.52 | 0 | 5 | 5 | 0.75 | 15 | 0.06 | 0 | 2 | 8 | 0.90 |
|  |  | 161 | 0.64 |  |  |  |  | 14 | 0.06 |  |  |  |  |
